# Co-Creative Vermittlungsplattformen für die Psychische Gesundheitsversorgung

**DOI:** 10.1365/s40702-022-00925-1

**Published:** 2022-11-03

**Authors:** Felix Kegel, Maike Greve, Knut Schnell, Miriam Stein, Lutz M. Kolbe

**Affiliations:** 1 University of Göttingen, Göttingen, Deutschland; 2 Asklepios Fachklinikum Göttingen, Universitätsmedizin Göttingen, Göttingen, Deutschland

**Keywords:** Psychische Gesundheitsversorgung, Gesundheits-IT, Co-Creation, Plattformentwicklung, Mental health care, Health IT, Co-creation, Platform development

## Abstract

Im deutschen Gesundheitswesen nimmt die Versorgung psychischer Störungen eine immer wichtigere Rolle ein. Nicht erst seit dem Beginn der Corona-Pandemie steigt die Zahl der Menschen mit psychischen Erkrankungen deutlich an. Damit gehen sowohl wirtschaftliche als auch persönliche Herausforderungen einher.

Dieser Artikel leistet einen Beitrag zur digitalen Transformation im Gesundheitswesen, indem eine konzeptionelle Analyse der Vermittlungsproblematik in der psychischen Gesundheitsversorgung vorgenommen wird. Die Studie zeigt, dass digitale Plattformen die Möglichkeit bieten, den bisherigen undurchsichtigen Angebotsmarkt zu strukturieren, Betroffenen sowie deren Angehörigen passende online und offline Versorgungs- und Unterstützungsangebote niederschwellig zugänglich zu machen und die Patient*innenreise sinnvoll zu erweitern. Die Ergebnisse verdeutlichen die Notwendigkeit der Berücksichtigung der Eigenschaften *Regionalität, Inklusion* und *Kollaboration* der Plattform sowie die der Umsetzung mit Hilfe co-creativer Methodiken. Zusammenfassend stellt die Studie eine umfassende und kontextualisierte Konzeption einer Vermittlungsplattform dar, und liefert damit sowohl für den wissenschaftlichen Diskurs im Bereich Gesundheits-IT als auch für Stakeholder aus der Praxis relevante Anhaltspunkte für die Gestaltung und Konzeption zukünftiger Plattformen.

## Abstract (en)

In the German healthcare system, the treatment of patients with mental disorders is taking on an increasingly important role. The number of people with mental illnesses in Germany has been rising significantly, and not just since the onset of the Covid-19 pandemic. This is accompanied by both economic and personal challenges. In this article, we contribute to the digital transformation in the healthcare sector by providing a conceptual analysis of the referral problem in mental health. To this end, we show that digital platforms offer a wide set of opportunities to structure the currently opaque supply side of the market and make both offline and online care and support services more accessible to patients and their relatives in a straightforward way, thus meaningfully enhancing the patient journey. Our results illustrate the necessity of considering the characteristics *regionality, inclusion* and *collaboration* of the platform and the implementation by means of co-creative techniques. In summary, our study presents a comprehensive and contextualized conceptualization of a referral platform and provides relevant evidence for the design and development of future platforms for both the academic discourse in the field of health IT and stakeholders from the field.

## Einleitung

Die Zunahme der direkten und indirekten Kosten psychischer Erkrankungen wird weltweit auf 6 Mrd. US-Dollar für das Jahr 2030 prognostiziert (Zylka-Menhorn 2011). Psychische Erkrankungen spielen laut der Organisation für wirtschaftliche Zusammenarbeit und Entwicklung die Hauptrolle bei Erwerbsminderungen und Frühberentungen. In Deutschland fehlt angesichts der hohen Zahl psychischer Erkrankungen (Jachertz 2013), allen voran Depressionen mit ca. 6 Mio. Betroffenen, häufig eine effektive Behandlung im häuslichen Alltag (Schnell und Herpertz 2011). Die Folge dieser Lücke in der Behandlung ist eine Verlängerung der Erkrankungsdauer, des subjektiven Leidens, der Arbeitsunfähigkeit und des sozialen Invalidierens. Vor diesem Hintergrund nimmt die adäquate Versorgung psychischer Störungen im deutschen Gesundheitssystem eine immer wichtigere Rolle ein. Dem hohen Bedarf an passenden Versorgungsangeboten wird auch mit der Entwicklung von immer neuen digitalen Therapieangeboten begegnet. Neben den digitalen Gesundheitsanwendungen (DiGAs), die als „Apps auf Rezept“ von Ärzt*innen und psychologischen Psychotherapeut*innen verschrieben werden können, gibt es zahlreiche Apps und Angebote von Krankenkassen, die zunehmend schwer zu überblicken sind.

Dieser Entwicklung steht die oftmals störungsbedingt eingeschränkte Informationsverarbeitung bei Patient*innen mit psychischen Störungen gegenüber, sodass eine Navigation durch die zahlreichen Angebote unmöglich wird. Auch die Angehörigen tragen bereits eine so hohe Versorgungslast, dass sie die Betroffenen nicht mehr ausreichend bei der Suche nach passenden Angeboten unterstützen können. Infolgedessen ist in der Praxis häufig zu beobachten, dass Versorgungsangebote erst spät oder gar nicht zu finden sind und insbesondere die digitalen Angebote noch wenig genutzt werden.

Auf der Basis dieser Beobachtungen rückt die Frage nach einer effektiven und effizienten Vermittlung individuell passender Behandlungsangebote an Betroffene in den Fokus. Dieser Frage kann mit der Entwicklung digitaler Plattformen begegnet werden. Der vorliegende Artikel definiert Vermittlungsplattformen für die psychische Gesundheitsversorgung als solche Plattformen, die Betroffene mit psychischen Erkrankungen niederschwellig dabei unterstützen, einen direkten und schnellen Zugang zu passenden Angeboten in der Versorgungslandschaft zu finden. Diese Plattformen können dabei sowohl die direkte Vermittlung passender Therapieangebote als auch die Weiterleitung an adäquate Beratungs- und Unterstützungsstellen sowie lokal aktive Peer-to-Peer Gruppen übernehmen, indem regional relevante Angebote gebündelt zugänglich gemacht werden. Allerdings ist die Verbreitung solcher digitaler Vermittlungs- und Austauschplattformen innerhalb der deutschen Versorgungslandschaft bislang noch nicht weit fortgeschritten. Neben der angebotsseitigen Unübersichtlichkeit im Markt fehlt es bei den Lösungen nicht selten an direkten Interaktions- bzw. Beitragsmöglichkeiten, sodass die Bedürfnisse der Betroffenen oft nur unzureichend in die Gestaltung der jeweiligen Angebote mit einfließen.

Dieser Artikel stellt die Hypothese auf, dass partizipative Gestaltungsansätze notwendig sind, um die beschriebenen Herausforderungen in der psychischen Gesundheitsversorgung mit Hilfe einer digitalen Vermittlungs- und Austauschplattform zu adressieren. Hierbei stehen drei unerlässliche Eigenschaften im Vordergrund: (1) Regionalität, die sich durch Einnahme einer Versorgungsperspektive begründen lässt; (2) Inklusion, die sowohl patient*innenseitig aus der eingeschränkten Informationsverarbeitung als auch aus dem insgesamt in der psychischen Gesundheitsversorgung anzuwendenden Grundprinzip des Trialogs resultiert (Einbeziehung aller relevanten Stakeholder: Patient*innen, Behandelnde, Angehörige); und (3) Kollaboration, welche ebenfalls im Trialogprinzip begründet ist. Durch die Kombination dieser Eigenschaften können digitale Vermittlungsplattformen eine Orientierung bieten, um relevante Angebote hinsichtlich ihrer Wirksamkeit, Zielgruppe und Zugänglichkeit zu filtern und übergreifend als digitale Wegweiser zu fungieren, um je nach Situation der Betroffenen die passenden Hilfsangebote zu identifizieren.

Im Folgenden wird diskutiert, wie sich das Problem des unzureichenden Zugangs zur psychischen Gesundheitsversorgung angebots- und nachfrageseitig manifestiert. Anschließend werden partizipative Gestaltungsmethoden und Co-Creation im Kontext von Gesundheitsforschung und -versorgung, sowie von digitalen Plattformen vorgestellt. Schließlich wird als mögliche Lösung eine Co-Creative Gesundheitsplattform zur Versorgung psychischer Störungen aufgezeigt und vor dem Hintergrund der drei zentralen Kriterien diskutiert. Somit leistet dieser Artikel einen Beitrag zur Debatte um die Potenziale von Gesundheits-IT zur Verbesserung der psychischen Gesundheitsversorgung in Deutschland und bietet eine Orientierung für entsprechende Design- und Implementierungsprojekte. Dies liefert damit sowohl einen Beitrag für den wissenschaftlichen Diskurs im Bereich digitale Transformation im Gesundheitswesen als auch für Stakeholder aus der Praxis.

## Das Vermittlungsproblem in der psychischen Gesundheitsversorgung

Bei der Suche nach einem passenden und verfügbaren Versorgungsangebot für Betroffene psychischer Störungen ergeben sich im Vergleich zur Informationssuche über somatische Erkrankungen einige spezifische Hindernisse. Diese Herausforderungen auf dem Weg zum passenden Versorgungsangebot manifestieren sich sowohl angebotsseitig als auch nachfrageseitig und werden im Folgenden dargelegt.

**Abb. 1:**
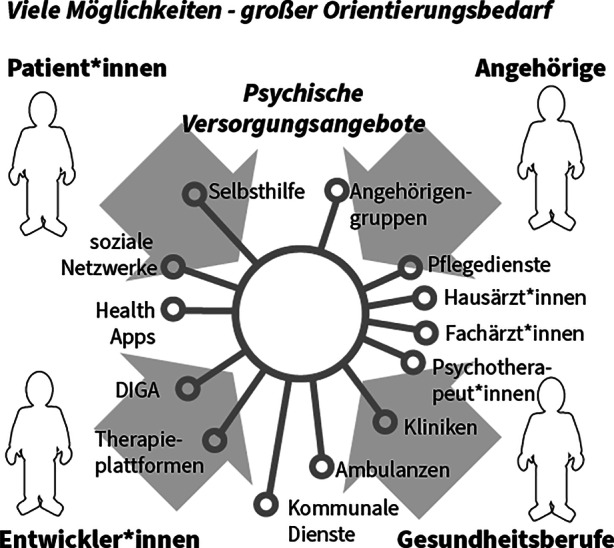
Der undurchsichtige Angebotsmarkt als Problem in der psychischen Gesundheitsversorgung

Zum einen bestehen auf der Seite der Behandler*innen zumindest in Deutschland Defizite hinsichtlich des Kenntnisstandes zu e‑Mental-Health-Angeboten und zu deren Integration in die eigene Behandlung. Die Hausärzt*innen, denen eine Lotsenfunktion im Gesundheitssystem zukommt, sind oftmals überfordert, angesichts der Fülle von neuen – auch digitalen – Versorgungsoptionen die immer aktuelle Übersicht zu behalten, um so ein individuell passendes Angebot oder auch eine Kombination aus Angeboten identifizieren zu können (Deutsches Ärzteblatt 2021; Lau 2022). Das Problem der mangelnden Informationsvermittlung über die digitalen Behandlungsmöglichkeiten für psychische Störungen spiegelt sich offensichtlich auch in der sehr zögerlichen ärztlichen und psychotherapeutischen Verordnung nachweislich wirksamer DiGAs wider: zwar steigen die Verordnungs- und Nutzungszahlen seit Einführung der DiGA im Oktober 2020 kontinuierlich an, allerdings ist das absolute Niveau dieser Zahlen weiterhin niedrig – dies gilt ganz besonders für DiGAs im Bereich der psychischen Gesundheitsversorgung (Techniker Krankenkasse 2022), die von Ärzt*innen und Psychologischen Psychotherapeut*innen verschrieben werden können. Schließlich stellt auch die sehr unübersichtliche Marktsituation mit Angeboten zur Förderung der psychischen Gesundheit eine große Hürde für deren intensivere Nutzung dar. Dies betrifft sowohl Angebote von medizinischen und nicht-medizinischen Stakeholdern im Markt als auch eine Reihe isolierter Einzellösungen, die separat von den Krankenversicherungen angeboten werden. Diese Unübersichtlichkeit am Markt (siehe Abb. 1) ist für Angehörige, aber gerade für die Patient*innen selbst ein häufig kaum zu überwindendes Hindernis bei der Suche nach Unterstützungs- und Behandlungsangeboten.

Gleichzeitig führt auf der Seite der Patient*innen die noch immer zu beobachtende Stigmatisierung psychischer Erkrankungen – wie z. B. die das Vorurteil „psychisch krank = gefährlich/faul etc.“ – zu einer verminderten Bereitschaft der Betroffenen, offen nach Unterstützungsmöglichkeiten zu fragen. Darüber hinaus kann nach wie vor eine Unterschätzung der inzwischen sehr gut entwickelten Behandlungsmöglichkeiten beobachtet werden: So werden etwa sowohl in der allgemeinen Öffentlichkeit als auch unter Behandelnden die Erfolgsaussichten psychiatrisch-psychotherapeutischer Behandlungen als geringer eingeschätzt als erwiesen (Gaebel et al. 2004). Die bereits dargestellte unübersichtliche Marktsituation trifft hier zudem auf eine oftmals gestörte Fähigkeit zur Informationsverarbeitung bei Patient*innen. Störungen der Fähigkeiten zur Informationsverarbeitung sowie der Motivation im Rahmen schwerer bzw. komplexer psychischer Erkrankungen erhöhen gerade für Betroffene mit dem höchsten Bedarf die Schwelle zur Nutzung digitaler Systeme. So zählen beispielsweise kognitive Defizite wie Aufmerksamkeitsstörungen und Probleme mit der internen Informationsverarbeitung zu gut erforschten Symptomen schizophrener Psychosen (Braff 1993; Carter et al. 2010; Fioravanti et al. 2005; Gebreegziabhere et al. 2022). Ähnliche Symptome können bei Patient*innen mit diagnostizierter bipolarer affektiver Störung (Bora und Pantelis 2015) oder schwerer Depression (Dehn und Beblo 2019; Rock et al. 2014) beobachtet werden, und schränken so die Möglichkeiten der Nutzung herkömmlicher digitaler Plattformen ein. Ein weiterer Faktor, der der großflächigeren Verbreitung von Online-Angeboten entgegensteht, ist, dass diese häufig als weniger nützlich als Face-to-Face-Dienstleistungen wahrgenommen werden (Apolinário-Hagen et al. 2017), was bedeutet, dass die Patient*innen sich primär an die Haus- und Fachärzt*innen wenden. Somit sind für diese Gruppen Fragen nach den passenden Angeboten, dem direktesten Zugang und auch der Kostenübernahme durch Versicherungen immer schwerer umfänglich zu beantworten.

Obige Ausführungen unterstreichen, dass niederschwellige Vermittlungsplattformen für die Versorgung psychischer Störungen mehr denn je gebraucht werden. Grundsätzlich bieten Informationssysteme vielversprechende Möglichkeiten, die existierenden Vermittlungsprobleme zu lösen. So könnten optimal designte Vermittlungsplattformen unter anderem dazu dienen, neben dem Aufzeigen passender Therapiemöglichkeiten physische Versorgungsangebote zu ergänzen oder die Überbrückung mehrmonatiger Wartezeiten auf ambulante Therapieplätze zu erleichtern. Auch nach dem Finden eines ersten passenden Behandlungsangebots hätten solche Plattformen einen erheblichen Nutzen, da sie die Patientenreise über psychiatrisch-psychotherapeutische Angebote hinaus, beispielsweise um weitere Bausteine wie Psychosozial- oder Familienberatung sowie den Zugang zu Selbsthilfegruppen, erweitern könnten. Aus Sicht der Behandelnden ergäben sich weitere Vorteile, etwa die Information über das regionale Ökosystem psychosozialer Unterstützungsangebote, mit deren Hilfe das eigene Behandlungskonzept sinnvoll ergänzt werden könnte.

Das Potenzial von Informationssystemen für die Vermittlung von Beratungs- und Behandlungsangeboten für die psychische Gesundheitsversorgung reiht sich dabei nahtlos in eine generelle Beobachtung ein, die seit Beginn der Corona-Pandemie über das Gesundheitssystem getroffen werden kann: Digitale Lösungen werden grundsätzlich stärker erforscht und eingesetzt als vor der Pandemie. Das gilt sowohl für jene Dienste, die spezifisch zur direkten Pandemiebekämpfung entwickelt wurden, wie bspw. Kontaktverfolgungsapps (vgl. Abeler et al. 2020; Munzert et al. 2021), als auch für solche, deren Einsatz die Pandemie verstärkt nötig gemacht hat, etwa verschiedene Telemedizinanwendungen und -plattformen (vgl. Tobias und Spanier 2020; Bahl et al. 2020). Auch in der psychischen Gesundheitsversorgung kommen digitale Angebote inzwischen häufig zum Einsatz (vgl. McGorry et al. 2022; Lehtimaki et al. 2021) und werden mehr und mehr erforscht (vgl. Sun et al. 2021).

Sämtliche oben genannten Punkte verdeutlichen die Notwendigkeit, sich näher und spezifisch mit der Gestaltung und dem Einsatz informationstechnischer Systeme zur Verbesserung der psychischen Gesundheitsversorgung auseinanderzusetzen. Einerseits müssen vorhandene Versorgungsangebote und relevante Informationen hierzu erheblich besser gebündelt und strukturiert werden. Um dies zu erreichen, sollten die Angebote nicht nur bundes- oder landesweit an zentraler Stelle gesammelt werden – es bietet sich vielmehr an, zugunsten der Nutzerfreundlichkeit die Vermittlungsplattformen auf bestimmte Regionen zu beschränken. So kann den Betroffenen ein unmittelbarer Zugang zu Angeboten in ihrer Nähe verschafft werden. Zwar ist auch eine Strukturierung auf Bundesebene grundsätzlich denkbar. Allerdings bestehen in der deutschen Versorgungslandschaft erhebliche regionale Unterschiede hinsichtlich der Facharztverfügbarkeit^1^ (siehe auch Tab. 1). Daraus ergeben sich wiederum regionsspezifische Bedarfe an Versorgungsangebote, die über regionale Plattformen besser abzubilden und zu bedienen sind. Gleichzeitig ist es essenziell, in die Gestaltung der nötigen Informationssysteme alle betroffenen Gruppen, wie beispielsweise Peer-to-Peer Gruppen, Partner*innen oder Kinder von Menschen mit psychischen Erkrankungen, einzubeziehen, um alle Interessen und Bedarfe ausreichend abbilden und bedienen zu können. Entsprechend liegt zur Entwicklung adäquater Informationssysteme für die Vermittlung passender Versorgungsleistungen die Verwendung partizipativer Gestaltungsansätze nahe.

**Tab. 1:** Übersicht über nötige Eigenschaften digitaler Vermittlungsplattformen für die psychische Gesundheitsversorgung

Beobachtungen	Abgeleitete Kerneigenschaft digitaler Plattformen
Regionale Unterschiede im Versorgungsangebot erfordern regionsspezifische kompensatorische und ergänzende Angebote^a^	Regionalität
Versorgungsregionen sind als Grundprinzip der psychischen Gesundheitsversorgung im deutschen Gesundheitswesen verankert (Gühne et al. 2019)	
Sicherstellung der Aktualität der dargestellten Informationen auf Bundesebene ist herausfordernd^b^	
Einfache Nutzung und Verständnis werden von Nutzenden erwartet (Cheng et al. 2021)	Inklusion
Patient*innen finden bisher erst zu spät (d. h. in chronifizierten Krankheitsstadien) zu Versorgungsangeboten^c^	
Förderung von Teilhabe in Subgruppen von Betroffenen hat positive Effekte, bspw. bei Einbeziehung kulturell diverser Populationen (Cheng et al. 2021)	Kollaboration (bei initialer *und* kontinuierlicher Weiterentwicklung)
Höhere Akzeptanz unter Nutzenden zu erwarten, wenn Betroffene als Expert*innen in den Entwicklungsprozess einbezogen werden (Sockolow et al. 2017)	

^a^Vgl. https://gesundheitsdaten.kbv.de/cms/html/16402.php: Große Varianz bzgl. regionaler Facharztverfügbarkeit

^b^Vgl. https://arztsuche.116117.de/pages/arztsuche.xhtml: Veraltete Informationen bzgl. Therapeut*innen in der Gesundheitsregion (Südniedersachsen) auf bundesweiten Webseiten

^c^https://www.thieme.de/de/psychiatrie-psychotherapie-psychosomatik/inklusion-psychisch-erkrankter-62435.htm

## Partizipative und co-creative Gestaltung von Gesundheits-IT

Partizipative Gesundheitsforschung eignet sich hervorragend dazu, die beschriebene Problemstellung im Rahmen eines inklusiven Forschungs- und Gestaltungsprozesses zu adressieren, da diese an dem grundsätzlichen Prinzip der Co-Creation ausgerichtet ist, dass sich alle involvierten Gruppen auf Augenhöhe begegnen, um Ideen zu generieren und weiterzuentwickeln (Wright 2021). Co-Creation-Ansätze haben ein hohes Potenzial für gesellschaftliche Auswirkungen, hängen aber entscheidend von Erfolgsprinzipien ab, wie beispielsweise von der iterativen Nutzer*innenevaluation und umfassenden Einbindung verschiedenster Stakeholder unter Berücksichtigung ihrer Rollen und Funktionen im gesamten Gesundheitsökosystem (Pouloudi et al. 2016).

Auch und gerade zur Versorgung psychischer Störungen ist das Anwenden co-creativer Methoden sinnvoll, um die zu entwickelnden Angebote passgenau auf die Bedürfnisse aller Beteiligten abstimmen und unmittelbar mit den relevanten Stakeholder-Gruppen erproben zu können. Ein solcher Fokus auf die nutzer*innenzentrierte Entwicklung kann beispielsweise dafür nützlich sein, eine inklusive Informationsergonomie zu schaffen, und somit den oben beschriebenen Problemen der Informationsverarbeitung bei Betroffenen mit spezifischen (schweren) psychischen Erkrankungen zu begegnen. Ihren Angehörigen kann durch diese Entwicklungsansätze zudem die Hilfestellung erleichtert und bei erfolgreicher Implementierung ein erheblicher Teil der Versorgungslast abgenommen werden. Dabei kann bereits im Gestaltungsprozess eine regionale Eingrenzung nützlich sein, um die relevanten Stakeholder möglichst unkompliziert zueinander zu bringen.

Die Berücksichtigung aller vorhandenen Stakeholder-Interessen mittels Co-Creation findet sich bereits in einem Kernprinzip zeitgemäßer psychosozialer Versorgungskonzepte, dem Trialog, wieder. Trialogformate zielen darauf ab, eine diverse Gruppe von Stakeholdern auf Augenhöhe miteinander interagieren zu lassen und sich über Erfahrungen und Bedürfnisse austauschen zu lassen, um Erkenntnisse sowohl zur Steuerung individueller Behandlungsabläufe als auch für die Gestaltung von Versorgungsstrukturen zu gewinnen. So werden beispielsweise Patient*innen, Angehörige und professionell Tätige zusammengebracht (Amering et al. 2012), was sich positiv auf die Reduzierung von Stigmatisierung und Diskriminierung auswirken kann (Mac Gabhann und Dunne 2021). Zudem werden das Finden einer gemeinsamen Sprache sowie die Fähigkeiten zur Perspektivübernahme der Beteiligten gefördert, wodurch das gegenseitige Verständnis gefördert wird. Angesichts dieser Vorteile sind trialogische Formate inzwischen integraler Bestandteil in der psychischen Gesundheitsversorgung und als solcher auch in der nationalen S‑3 Leitlinie „Psychosoziale Therapien bei schweren psychischen Erkrankungen“ verankert (Gühne et al. 2019). Eng verwoben ist der Trialog darüber hinaus mit dem Begriff des Empowerment, welcher die Fähigkeit von Menschen beschreibt, sich frei entwickeln, ihr Leben den eigenen Vorstellungen entsprechend gestalten und ihre Bedürfnisse auch anderen gegenüber durchsetzen zu können (Gühne et al. 2019). Dabei setzt auch erfolgreiches Empowerment die aktive Beteiligung aller relevanten Stakeholder voraus.

## Drei Säulen der Gestaltung co-creativer Vermittlungsplattformen zur psychischen Gesundheitsversorgung

**Abb. 2:**
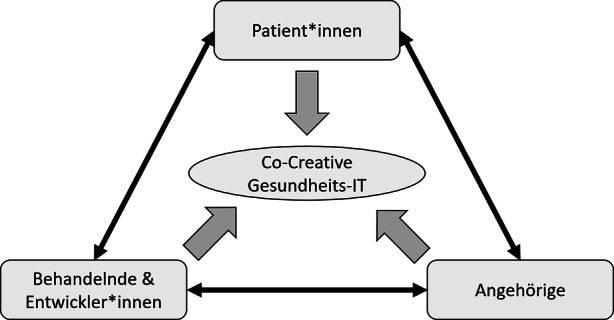
Einzubeziehende Nutzer*innen im Sinne des Trialogs

Hauptsächlicher Beitrag dieses Artikels ist die Verdeutlichung des Nutzens co-creativer Ansätze für die IT-Gestaltung im Bereich der psychischen Gesundheitsversorgung. Wie oben diskutiert sollten partizipative Methoden für das Design und die Implementierung von Plattformen verwendet werden, um die Vermittlungs- und Versorgungsprobleme zu adressieren. Die grundsätzlich zu beteiligenden Gruppen sind – basierend auf dem Prinzip des Trialogs – in Abb. 2 dargestellt. Die Literatur weist darauf hin, dass Positionen mehrerer Interessengruppen miteinander verflechten und dass sogar dieselbe Fokusgruppe konkurrierende Positionen einnehmen kann, die die Akzeptanz und Nutzung der Gesundheits-IT untergraben (Pouloudi et al. 2016). Deswegen ist die partizipative Einbindung durch Co-Creation-Methodiken essenziell.

Bei der Konzeption ist dann insbesondere auf die folgenden drei Eigenschaften zu achten, die sich in den zu entwickelnden Systemen wiederfinden sollten und die sich aus diversen Beobachtungen in Forschung und Praxis ergeben (vgl. Tab. 1):

- *(1) Regionalität.* Eine geographische Festlegung, beispielsweise auf bestimmte Gesundheitsregionen, erscheint sinnvoll, um entstehende Plattformen in ihrer Komplexität zu beschränken und somit übersichtlich zu gestalten. Wenn psychiatrisch-psychotherapeutische Versorgungsangebote für Betroffene auch räumlich leicht zu erreichen sind, kann in der Wahrnehmung von Patient*innen von einer deutlichen Nutzensteigerung ausgegangen werden. Der Zugang zu passenden Beratungs- und Versorgungsdienstleistungen wird somit direkter und räumliche Zugangsbarrieren per Design verringert. Dadurch können digitale Plattformen im Rahmen des regionalen Bezugs insbesondere die Unterstützungsmöglichkeiten durch einen direkten menschlichen Kontakt in Therapie‑, Präventions- und Selbsthilfeangebote vor Ort identifizieren und zugänglich machen. Das Interesse dieser Stakeholder daran, gefunden zu werden, motiviert zur Mitarbeit an der inkrementellen Weiterentwicklung und sorgt somit für eine kontinuierliche Verbesserung der Plattformen. Darüber hinaus ist davon auszugehen, dass sich die Grundzüge dieser regionalen Informationssysteme unkompliziert auf andere Gesundheitsregionen übertragen lassen, da sich die grundsätzlichen Probleme in der Versorgung psychischer Störungen und der entsprechenden Vermittlung der Betroffenen bundesweit ähneln. Somit kann nach erfolgreichen, regionalen Testphasen auch die weiträumigere Verbreitung der Systeme adressiert und angestoßen werden.
- *(2) Inklusion*. Der niederschwellige Zugang zu den Systemen sollte für alle Beteiligten in mehrfacher Hinsicht gewährleistet werden. Einerseits muss während des gesamten Design-Vorgangs darauf geachtet werden, alle relevanten Stakeholder-Gruppen (siehe Abb. 2) als gleichwertige Partner*innen eines co-creativen und inkrementellen Entwicklungsprozesses zu betrachten und zu beteiligen. So werden der wahrgenommene Nutzen sowie die Akzeptanz der Systeme gesteigert (Fortuna et al. 2019). Dies kann beispielsweise durch qualitative Forschungsmethoden wie Interviews oder Fokusgruppen oder aber durch gemeinsame, co-creative Gestaltungsworkshops erreicht werden. Ebenso können sich trialogähnliche Formate in der Konzeption der Plattformen bewähren, wenn die Interessen aller Gruppen berücksichtigt werden und entsprechend die nutzer*innenzentrierte Entwicklung im Fokus steht. Andererseits muss auch das zu entwickelnde System für alle Nutzenden leicht zugänglich und bedienbar sein. Dies gilt ganz besonders aus der Perspektive der Patient*innen, deren Informationsverarbeitung wie beschrieben häufig eingeschränkt ist. Dabei kann zum Beispiel das User Interface adaptiv gestaltet sein, sodass unterschiedlichen Nutzer*innen je nach Rolle unterschiedliche Aktionsmöglichkeiten angeboten und Informationen dargestellt werden. So ist etwa eine Plattform denkbar, in deren Gestaltungsprozess von Anfang an darauf hingearbeitet wird, die User Experience auf zwei differenzierte Nutzer*innenreisen anzupassen: eine verstärkt niederschwellige Variante, die für Patient*innen selbst optimiert ist, d. h. mit sehr übersichtlicher Darstellung, verständlicher Sprache und möglichst direkten Vermittlungsmöglichkeiten; dagegen wäre zum Beispiel für Angehörige und Behandelnde eine andere User Experience vorstellbar, da ihre Informationsverarbeitungsfähigkeiten die Verarbeitung komplexerer Informationen und Zusammenhänge zulassen.
- *(3) Kollaboration.* Weiterhin ist es essenziell, sich auch auf die in den Systemen angebotenen Inhalte und Vermittlungsdienstleistungen selbst zu konzentrieren. Es sollte ein Hauptaugenmerk darauf gelegt werden, mehr anzubieten als eine bloße Auflistung oder Datenbank aller in der Region verfügbaren Versorgungs- und Unterstützungsangebote, die mit Filter- oder Suchfunktionen passend einzuschränken sind. Vielmehr kann ein adäquates Design der Plattform selbst zur Nutzer*innenbeteiligung anregen, etwa durch die Möglichkeit, dass Patient*innen und Angehörige eigene Beiträge, als Erfahrungsberichte, Ankündigungen, oder Änderungsvorschläge für die Gestaltung der Plattform selbst und deren inkrementelle Weiterentwicklung, im Rahmen von Redaktionsteams oder Workshops, einbringen können. Ebenso ist es denkbar, dass Behandelnde Angebote, die über klassische psychiatrisch-psychotherapeutische Behandlungsleistungen hinausgehen, beispielsweise zu co-creativen Workshops oder Trialogformaten direkt in die Systeme einbinden, sodass sich interessierte Nutzer*innen unkompliziert anmelden können. Weitere Funktionen könnten auf die Integration von Austauschformaten abzielen, die mitunter keine Begleitung professionell tätiger Versorgungsdienstleister benötigen. So werden in den letzten Jahren beispielsweise soziale Netzwerke wie Facebook, Twitter und Youtube immer häufiger von Betroffenen genutzt, um sich untereinander auszutauschen und so das eigene Empowerment zu fördern (Naslund et al. 2016). Solche Peer-to-Peer-Ansätze sind in der psychischen Gesundheitsversorgung bereits etabliert (etwa über Selbsthilfegruppen) und können entsprechend als (digitale) Ergänzung zu physischen Face-to-Face-Angeboten wichtige Beiträge zur Verbesserung der Versorgungslage darstellen. Dabei ist trotz der zahlreichen Vorteile partizipativer Formate und Entwicklungsmethoden darauf zu achten, dass die eingestellten Inhalte kontinuierlich gemeinsam überprüft werden. Möglichkeiten dazu sollte es sowohl initial bei Einbindung in die Vermittlungsplattform als auch danach durch die Nutzer*innen geben, um die kritischen Inhalte flaggen können.

**Abb. 3:**
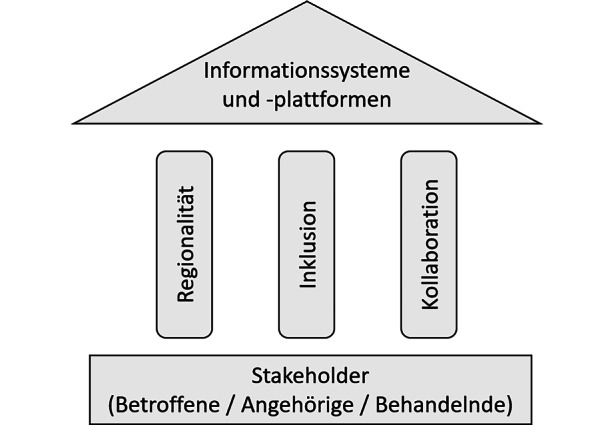
Werte und Faktoren zur Gestaltung co-creativer Gesundheitsplattformen

Angelehnt an diese drei zentralen Werte zeigt Abb. 3 die konzeptionelle Ausrichtung co-creativer Gesundheitsplattformen zur Versorgung psychischer Störungen. Fundamental ist dabei die partizipative Gestaltung und Einbindung der Stakeholdergruppen. Aufbauend darauf setzen die drei beschriebenen Eigenschaften auf, wodurch eine digitale Plattform ein verbindendes Element für einen nachhaltigen und essenziellen Lösungsansatz in der Vermittlungsproblematik darstellen kann.

Um die Potenziale digitaler Vermittlungsplattformen für die psychische Gesundheitsversorgung zukünftig vollständig zu erschließen, müssen an dieser Stelle die Limitationen unserer Studie diskutiert werden. Einerseits ist es denkbar, dass neben den oben genannten Haupteigenschaften *Regionalität, Inklusion* und *Kollaboration* noch weitere Merkmale relevant sind. Künftige Forschung könnte in dieser Hinsicht, bestenfalls mit Hilfe partizipativer Forschungsansätze versuchen, zusätzliche, im Kontext der psychischen Gesundheitsversorgung wichtige Kriterien zu identifizieren und in das Design informationstechnischer Vermittlungsplattformen einfließen zu lassen. Ebenso ist darauf zu achten, grundsätzlich relevante Designprinzipien für digitale Plattformen zu berücksichtigen. Dies beinhaltet sowohl die generelle Nutzerfreundlichkeit als auch die Aktualität der Plattforminhalte, die unabhängig vom Anwendungskontext essenziell sind. Darüber hinaus bedarf es einer Evaluation der hier abgeleiteten Eigenschaften in Form einer praxisnahen Umsetzung unter Einbeziehung der genannten relevanten Stakeholder. Nur so kann beurteilt werden, inwieweit eine auf den Prinzipien basierende Vermittlungsplattform auch tatsächlich die im Artikel diskutierten Problematiken adressieren und beheben kann. Eine solche Umsetzung und Evaluation hat bislang nicht stattgefunden, befindet sich aktuell jedoch in einer ersten Iteration im Rahmen eines co-creativen Workshops in Q3 2022. Es ist davon auszugehen, dass solche Veranstaltungen weitere relevante Einblicke und Ergebnisse generieren können.

## Fazit

Dieser Artikel zeigt die Potenziale co-creativer Gestaltungsansätze für das Design von Vermittlungsplattformen für die psychische Gesundheitsversorgung auf. Er leitet hierzu ausführlich in die Problemstellung ein, dass die angemessene Vermittlung von Menschen mit psychischen Störungen zu Versorgungsangeboten im deutschen Gesundheitssystem durch mehrerlei angebots- und nachfrageseitig gegebene Herausforderungen behindert wird. Unter Bezugnahme auf die Grundprinzipien co-creativer Methoden, die sich in der Entwicklungs- und Versorgungspraxis etabliert haben, werden drei zentrale Eigenschaften abgeleitet, die innovative und adäquate informationstechnische Vermittlungsplattformen definieren sollten: *Regionalität, Inklusion*, und *Kollaboration*. Regionalität ist unerlässlich, um die inkludierten Behandlungs- und Unterstützungsangebote zu bündeln und die Marktsituation für die Nutzer*innen übersichtlich zu halten. Dagegen ist Inklusion sowohl im initialen als auch im inkrementellen, partizipativen Weiterentwicklungsprozess essenziell, um die Bedürfnisse aller involvierten Stakeholder angemessen zu adressieren. Und schließlich kann Kollaboration dazu dienen, Nutzer*innen zu aktiver Partizipation am Weiterentwicklungsprozess und zur Verbesserung der Patient*innenreise anzuregen, wodurch zusätzlich ein stärkeres langfristiges User Engagement zu erwarten ist.

Mit der Diskussion dieser Eigenschaften trägt der Artikel erheblich zur Debatte um die Einsatzmöglichkeiten von Informationssystemen im Gesundheitswesen bei und definiert Schwerpunkte, die in deren Entwicklungsprozess eine zentrale Rolle einnehmen sollten. Hierbei wird besonders das wachsende Angebot an digitalen Mental-Health-Angeboten adressiert, welches als zusätzliche Versorgungsform bisher nur limitierte Anwendung in der Praxis findet. Aus wissenschaftlicher Perspektive leistet dieser Artikel einerseits einen Beitrag zum Forschungsdiskurs im Bereich e‑Mental-Health, andererseits aber auch zum Diskurs bezüglich der kontextuellen Einsatzfelder für co-creative Methodiken. Dieser Artikel bewegt sich zwar noch auf konzeptioneller Ebene, bildet aber das Grundgerüst für die Entwicklung einer partizipativen Plattform für psychische Gesundheitsversorgung in der Gesundheitsregion Südniedersachsen, die mit einem ersten co-creativen Workshop in Q3 2022 mit verschiedene Stakeholdern den iterativen Entwicklungsprozess einer Vermittlungsplattform startet und im Zuge dessen die vorgestellten Gestaltungsansätze in der Praxis erprobt und ihren tatsächlichen Nutzen bewertet.

## Footnotes

1 https://gesundheitsdaten.kbv.de/cms/html/16402.php.
